# Nutritional Cognitive Neuroscience: Innovations for Healthy Brain Aging

**DOI:** 10.3389/fnins.2016.00240

**Published:** 2016-06-06

**Authors:** Marta K. Zamroziewicz, Aron K. Barbey

**Affiliations:** ^1^Beckman Institute for Advanced Science and Technology, University of Illinois at Urbana-ChampaignUrbana, IL, USA; ^2^Neuroscience Program, University of Illinois at Urbana-ChampaignChampaign, IL, USA; ^3^Carle R. Woese Institute for Genomic Biology, University of Illinois at Urbana-ChampaignChampaign, IL, USA; ^4^Department of Bioengineering, University of Illinois at Urbana-ChampaignChampaign, IL, USA; ^5^Department of Internal Medicine, University of Illinois at Urbana-ChampaignChampaign, IL, USA; ^6^Department of Psychology, University of Illinois at Urbana-ChampaignChampaign, IL, USA; ^7^Department of Speech and Hearing Science, University of Illinois at Urbana-ChampaignChampaign, IL, USA

**Keywords:** nutritional cognitive neuroscience, nutritional epidemiology, cognitive neuroscience, nutrient biomarkers, cognitive aging

## Abstract

Nutritional cognitive neuroscience is an emerging interdisciplinary field of research that seeks to understand nutrition's impact on cognition and brain health across the life span. Research in this burgeoning field demonstrates that many aspects of nutrition—from entire diets to specific nutrients—affect brain structure and function, and therefore have profound implications for understanding the nature of healthy brain aging. The aim of this *Focused Review* is to examine recent advances in nutritional cognitive neuroscience, with an emphasis on methods that enable discovery of nutrient biomarkers that predict healthy brain aging. We propose an integrative framework that calls for the synthesis of research in nutritional epidemiology and cognitive neuroscience, incorporating: (i) methods for the precise characterization of nutritional health based on the analysis of nutrient biomarker patterns (NBPs), along with (ii) modern indices of brain health derived from high-resolution magnetic resonance imaging (MRI). By integrating cutting-edge techniques from nutritional epidemiology and cognitive neuroscience, nutritional cognitive neuroscience will continue to advance our understanding of the beneficial effects of nutrition on the aging brain and establish effective nutritional interventions to promote healthy brain aging.

## Nutritional cognitive neuroscience and healthy brain aging

As the aged population expands, the economic burden of care and treatment of those with age-related health disorders also increases. Between 2012 and 2050, the United States will experience significant growth in its older population, with the size of the population aged 65 and over almost doubling from an estimated 43.1 million in 2012 to 83.7 million in 2050 (Ortman and Guarneri, 2009). Therefore, a successful strategy to promote healthy brain aging is of great interest to public health efforts and the United States economy. Diet and the many bioactive substances present in food represent a novel target for interventions that may promote healthy brain aging. Emerging evidence in nutritional cognitive neuroscience indicates that optimal nutrition may serve as a potential avenue to preserve cognitive function, slowing the progression of aging and reducing the incidence of debilitating diseases in healthy aging populations.

The aim of this *Focused Review* is to examine recent advances in **nutritional cognitive neuroscience**, with an emphasis on methods that enable discovery of nutrient biomarkers that predict healthy brain aging. We propose an integrative framework that calls for the synthesis of research in nutritional epidemiology and cognitive neuroscience, incorporating: (i) methods for the precise characterization of nutritional health based on the analysis of nutrient biomarker patterns (NBPs) along with (ii) modern indices of brain health derived from high-resolution magnetic resonance imaging (MRI) of brain structure and function. We begin by surveying recent methodological advances in nutritional epidemiology, followed by a review of contemporary methods for the neuroscientific study of brain aging. By integrating methods from nutritional epidemiology and cognitive neuroscience, the burgeoning field of nutritional cognitive neuroscience will continue to advance our understanding of the beneficial effects of nutrition on the aging brain and establish effective nutritional interventions to promote healthy brain aging.

KEY CONCEPT 1. Nutritional cognitive neuroscience Nutritional cognitive neuroscience is an interdisciplinary field of research that investigates the impact of nutrition on cognition and brain health across the life span. The aim of this Focused Review is to examine recent advances in nutritional cognitive neuroscience, specifically highlighting the utility of nutrient biomarkers in predicting healthy brain aging. Our proposed framework integrates research across nutritional epidemiology and cognitive neuroscience, combining: (i) methods for the precise characterization of nutritional status with (ii) modern measures of brain health.

## Methodological advances in nutritional epidemiology

Observational studies indicate that particular nutrients have beneficial effects on brain aging, including antioxidant nutrients, calcium, fiber, folate, zinc, omega-3 polyunsaturated fats, and vitamins A, B12, C, D, and E (Malinin et al., 2011; Mohajeri et al., 2015; Sheats et al., 2015). However, these findings have not been consistently replicated in randomized controlled trials (Wald et al., 2010; Dangour et al., 2012; Mazereeuw et al., 2012; Clarke et al., 2014; Massee et al., 2015). A primary source of inconsistency among findings is thought to reflect methodological limitations in the assessment of dietary patterns (Zuniga and McAuley, 2015), giving rise to new methods in nutritional epidemiology that examine blood biomarkers that are associated with healthy brain aging.

### Biochemical markers of dietary intake

Traditional research in nutritional epidemiology has examined food intake on the basis of self-reported dietary assessment methods such as food frequency questionnaires, 24-h recall, and weighed food records (Zuniga and McAuley, 2015). Although these methods can be implemented in large samples with relative ease, they are associated with measurement error. Primary sources of error include energy expenditure under-reporting, recall errors, and difficulty assessing portion sizes (Bingham, 2002; Kipnis, 2003). Furthermore, cognitive decline (e.g., memory loss) may limit recall on self-reported dietary assessments, and therefore bias nutritional assessment in older adults (Reuter-Lorenz and Park, 2010; Zuniga and McAuley, 2015). In addition, biases in self-reported dietary data are known to be influenced by age, gender, socioeconomic status, and education (Thompson and Subar, 2013). Finally, self-reported dietary assessment methods fail to account for variability in nutrient absorption (Scalbert et al., 2014).

Biochemical markers of dietary exposure have been developed to circumvent the measurement errors of dietary assessment techniques (Combs et al., 2013). Biomarkers can provide measures of nutritional status and exposure to bioactive molecules in foods, and thus can be used as surrogate indicators of food intake (Potischman and Freudenheim, 2003). Biomarker measurement also permits the identification of nutrient deficiencies and therefore allows treat-to-target paradigms, rather than global dietary approaches (Combs et al., 2013).

Biochemical markers can be analyzed from blood, urine, or tissue. The concentration of a given marker reflects intake of a particular dietary component (Jenab et al., 2009; Zuniga and McAuley, 2015). Epidemiological studies have identified approximately 100 biomarkers that correlate with dietary intake (Table 1; Scalbert et al., 2014). These biomarkers can be measured to estimate intake of a wide range of dietary components, including overall fruit and vegetable intake (Mennen et al., 2006; Baldrick et al., 2011), citrus fruits (Heinzmann et al., 2010; Lloyd et al., 2011a; Pujos-Guillot et al., 2013), cruciferous vegetables (Edmands et al., 2011; Andersen et al., 2014), salmon (Lloyd et al., 2011b), red meat (Stella et al., 2006; Cross et al., 2011), soy (Verkasalo et al., 2001), whole grain cereals (Andersson et al., 2011; Ross et al., 2012), coffee (Nagy et al., 2011; Rothwell et al., 2014), tea and wine (Hodgson et al., 2004; Mennen et al., 2006), food additives (Brantsaeter et al., 2009), and food contaminants (Turunen et al., 2010). As a complement to self-reported methods, biochemical analyses of nutrient biomarkers can improve data validity by providing an objective and sensitive assessment of a wide range of dietary components (Elmadfa and Meyer, 2014).

**Table 1:** **Biochemical markers of dietary exposure**.

**Vitamins**	**Fatty acids**
B1 (thiamine)	α –Linolenic acid
B2 (riboflavin)	Arachidonic acid
B5 (pantothenic acid)	cis-Docosapentaenoic acid
B6 (pyridoxine)	cis-Octadecenoic acid
B9 (folate)	cis-Palmitoleic acid
B12 (cobalamin)	Docosahexaenoic acid (DHA)
C	Docosapentaenoic acid (DPA)
D	Eicosapentaenoic acid (EPA)
E	Eicosadienoic acid
K1	Eicosenoic acid
Nicotinamide	Elaidic acid
**Carotenoids**	Lauric acid
α-carotene	Linolelaidic acid
β-carotene	Linoleic acid
β-cryptoxanthin	Myristic acid
Lutein	Myristoleic acid
Zeaxanthin	Oleic acid
Lycopene	Omega-3 polyunsaturated fats
**Polyphenols**	Omega-6 polyunsaturated fats
4-O-Methylgallic acid	Palmitic acid
5-Heneicosylresorcinol	Petroselaidic acid
5-Heptadecylresorcinol	Phytanic acid
5-Nonadecylresorcinol	Rumenic acid
5-Tricosylresorcinol	Stearic acid
Apigenin	Tetradecenoic acid
Caffeic acid	trans-Hexadecenoic acid
Chlorogenic acid	trans-Octadecadienoic acid
Daidzein	trans-Octadecenoic acid
DHBA	Vaccenic acid
DHPPA	**Amino acids**
Dihydrodaidzein	1-Methylhistidine
Dihydrogenistein	3-Methylhistidine
Enterodiol	**Organic compounds**
Enterolactone	Taurine
Equol	**Aliphatic acyclic compounds**
Eriodictyol	Urea
Gallic acid	**Chemical elements**
Genistein	Nitrogen
Glycitein	**Food contaminants**
Hesperetin	Aflatoxins
Isorhamnetin	Mercury
Kaempferol	PCBs
Luteolin	**Cooking products**
m-Coumaric acid	Acrylamide
Naringenin	1-Hydroxypyrene glucuronide
ODMA	**Endogenous metabolites and enzymes**
Phloretin	5-Hydroxytryptophol
Quercetin	ALAT
Resveratrol	ASAT
Tamarixetin	GGT
**Inorganic compounds**	
Iodine	
Phosphorous	
Potassium	
Selenium	
Sodium	
Zinc	
Iron	
Calcium	

### Holistic dietary patterns

Research in nutritional epidemiology has historically examined health outcomes in relation to one or a few nutrients. Although this type of analysis has been valuable, it has several conceptual and methodological limitations. First, rather than eating isolated nutrients, most individuals consume diets that consist of complex combinations of nutrients that have interactive effects. As a consequence, the single nutrient approach may be inadequate for taking into account interactions among nutrients. Second, the effect of a single nutrient may be too small to detect, but the cumulative effects of multiple nutrients included in a dietary pattern may be sufficiently large to be detectable. Finally, because nutrient intakes are commonly associated with certain dietary patterns, single nutrient analysis may potentially be confounded by the effect of dietary patterns. Dietary patterns represent a broader picture of food and nutrient consumption, and may thus be more predictive of cognitive function and brain health than individual foods or nutrients (Barberger-Gateau, 2014).

The importance of studying dietary patterns has become increasingly recognized in the scientific community, motivating an investigation of the role of specific dietary patterns in cognitive aging. A dietary pattern that has received significant attention is the Mediterranean (MEDI) diet (Willett et al., 1995; Trichopoulou et al., 2015). The MEDI diet is comprised of foods that are known to deliver beneficial nutrients, including olive oil that provides monounsaturated fats and polyphenols, fish that delivers omega-3 polyunsaturated fats and vitamin D, and fruits and vegetables that provide vitamins C and E, carotenoids, folate, and polyphenols (Sofi et al., 2013). Combinations of these nutrients may optimize the protective vascular, antioxidant, and anti-inflammatory mechanisms promoted by these nutrients (Sofi et al., 2013). Meta-analytic reviews provide evidence to support the efficacy of the MEDI diet, suggesting that this dietary pattern may have protective effects on cognitive aging (Sofi et al., 2013).

Recent studies further indicate that specific dietary patterns may have targeted effects. For example, the Dietary Approach to Stop Hypertension (DASH) diet, which consists of nutrient dense foods and low-sodium intake, is associated with reduced hypertension and improved psychomotor speed (The Seventh Report of the Joint National Committee on Prevention, Detection, Evaluation, and Treatment of High Blood Pressure, 2004; Smith et al., 2010). The promising effects of both the MEDI and DASH diets have motivated a fusion of these dietary patterns in the Mediterranean-Dietary Approach to Systolic Hypertension Diet Intervention for Neurodegenerative Delay (MIND) diet. The MIND diet is known to slow age-related cognitive decline in episodic memory, semantic memory, and perceptual speed (Morris et al., 2015). Thus, evidence indicates that the MEDI, DASH, and MIND diets may prevent or slow age-related changes in brain health, motivating the use of blood biomarkers to better characterize the effects of these dietary patterns on brain aging.

### Nutrient biomarker patterns

Scientific advances in the characterization of dietary patterns and the analysis of nutrient biomarkers have led to new methods in nutritional epidemiology for the measurement of **nutrient biomarker patterns (NBP)**. This approach applies Principal Component Analysis to capture the effects of nutrients in combination, enabling discovery of patterns of nutrient biomarkers. This method detects NBPs in plasma and therefore avoids methodological problems in traditional food frequency questionnaires, such as faulty recall of dietary intake and failure to account for variability in nutrient absorption (Scalbert et al., 2014). Each NBP represents a linear combination of individual plasma nutrients that load heavily within each biomarker pattern. Each participant receives a standardized NBP score for each pattern, and this score can subsequently be used to assess the relationship between nutrient patterns, cognitive function, and brain health. Early applications of this method have revealed multiple nutrient patterns that influence cognition and brain aging, including an NBP composed of antioxidants C and E, B vitamins, and vitamin D associated with enhanced global cognitive function; and an NBP consisting of omega-3 polyunsaturated fatty acids eicosahexaenoic acid (EPA) and docosahexaeonic acid (DHA) associated with white matter integrity (Bowman et al., 2012).

KEY CONCEPT 2. Nutrient biomarker patterns Modern methods in nutritional epidemiology provide an objective measure of nutritional status based on nutrient biomarker patterns derived from exploratory or hypothesis-driven analysis techniques.

Metabolomics provides a second approach to characterizing NBPs based on high-throughput analytic chemistry technologies that assess all small molecules associated with metabolism, known as the metabolome (Scalbert et al., 2014). The human metabolome is not a single entity—it consists of many components, including the endogenous metabolome, which represents cellular metabolism, the food metabolome, which reflects chemicals derived from digestion and metabolism of food, and xenobiotics acquired from the environment and drugs. Metabolomics provides the opportunity to investigate the complex interactions between dietary components, as well as between dietary components and the human body. This method allows researchers to measure hundreds to thousands of metabolites at a time (Scalbert et al., 2014). In doing so, biomarker panels common to particular foods or dietary patterns and the mechanistic effects of diet on metabolic pathways can be examined (Gibbons et al., 2015).

By characterizing individual dietary phenotypes with an unprecedented scope and level of precision, metabolomics can identify biomarkers of aging and elucidate the mechanisms of health status in an effort to improve early diagnosis, facilitate accurate prognosis, and assist in monitoring of patient response to therapy (Dunn et al., 2011). Metabolomics has identified potential biomarkers for a variety of foods and dietary patterns, including raspberries (Lloyd et al., 2011b), broccoli (Lloyd et al., 2011b), citrus fruits (Heinzmann et al., 2010), overall fruit and vegetable intake (O'Sullivan et al., 2011), high meat diets (O'Sullivan et al., 2011), and the Western diet (Bouchard-Mercier et al., 2013). Research applying metabolomics to investigate the relationship between the food metabolome and brain aging, however, remains at an early stage, with no published studies conducted to date (Zuniga and McAuley, 2015). Given the complexity of the food metabolome, validation of dietary markers is still underway (Scalbert et al., 2014), along with efforts to identify specific patterns within the food metabolome that are associated with healthy brain aging (Scalbert et al., 2014).

## Magnetic resonance imaging measures of brain aging

**Magnetic resonance imaging (MRI)** enables the study of structural and functional brain changes associated with aging and the prediction of neuropathological processes in the aging brain (Buckner, 2004). Even within cognitively normal brains, neurodegenerative processes can be present and measured using MRI (Wilson et al., 1999; Mungas et al., 2002; Rusinek et al., 2003). MRI methods therefore provide the foundation for investigating structural and functional changes in the aging brain and examining the impact of nutrition on healthy brain aging.

KEY CONCEPT 3. Magnetic resonance imaging Contemporary neuroscience methods provide measures of brain structure and function on the basis of high-resolution magnetic resonance imaging.

### Structural neuroimaging

**Figure 1:**
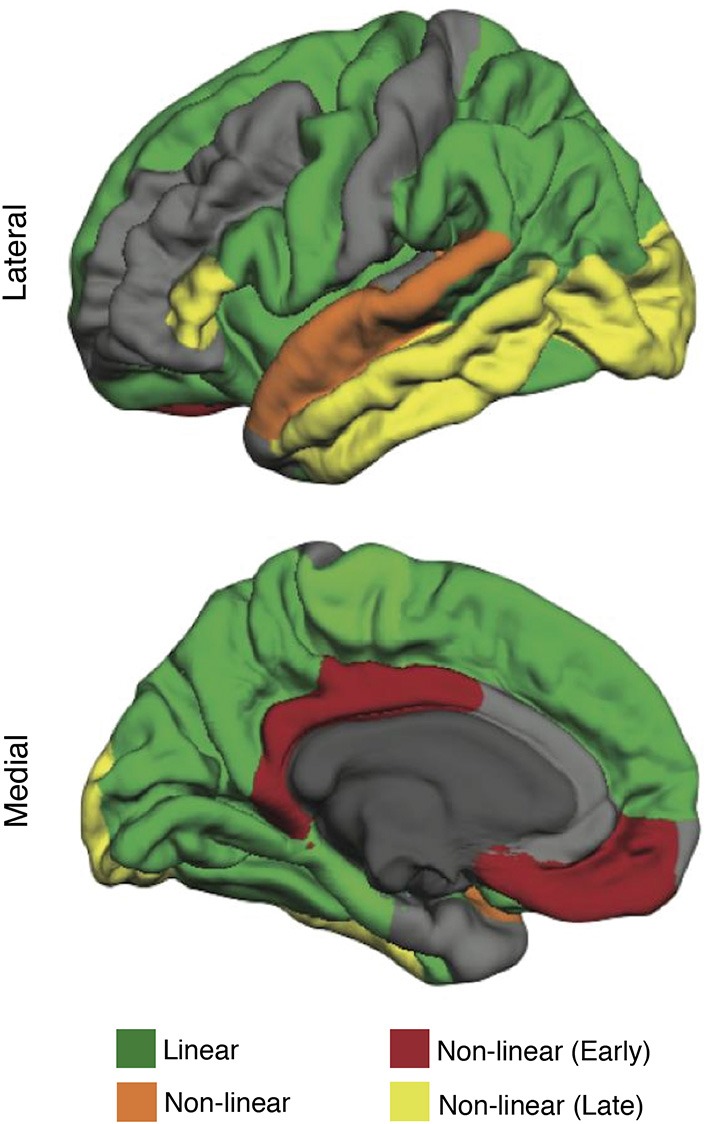
**The effect of aging on brain structure (cortical thickness) in healthy older adults (mean age 63.38 ± 12.23 years)**. Regions highlighted in green follow a linear rate of atrophy. Regions highlighted in orange show decline early in aging, stabilize, and then decline again late in aging. Regions highlighted in red show decline early in aging (decrease quickly early in aging but stabilize late in aging). Regions highlighted in yellow show decline late in aging (remain structurally intact early in aging but decrease quickly late in aging). Modified from Claassen et al. (2016) in Aging and Disease licensed under CC BY.

Structural MRI enables high-resolution imaging of age-related changes in gray and white matter structure, including: (1) total and regional brain volume (volumetry), (2) integrity of white matter fiber tracts (diffusion tensor imaging), (3) axonal microstructure of brain tissue (MR-elastography), and (4) altered mineral content (MR-gradient echo imaging) (Grady, 2000; Lockhart and DeCarli, 2014). Application of these methods has revealed the heterogeneous nature of brain aging. Although atrophy across the whole brain is evident with aging, these changes vary by region and tissue type. Differential effects of aging are particularly evident in the cerebral cortex, in which the superior frontal, middle frontal, and superior parietal cortex are most susceptible to steady age-related atrophy (Lockhart and DeCarli, 2014). Other cortical regions have fluctuating rates of change, with some areas showing accelerated atrophy early in aging, others demonstrating accelerated atrophy late in aging, and others showing a combination of early and late acceleration (Figure 1; Lockhart and DeCarli, 2014; Claassen et al., 2016). Subcortically, the caudate nucleus, cerebellum, and hippocampus show susceptibility to age-related structural degeneration (Raz et al., 2005). A particularly common age-related disruption to brain tissue is the deterioration of cerebral white matter, known as white matter lesions (Lockhart and DeCarli, 2014). White matter lesions are more extensive in individuals with cardiovascular risk factors; however, even borderline changes in blood pressure can result in white matter lesions (Longstreth et al., 1996; Swan et al., 1998). In addition to lesions, white matter also shows reduced microstructural integrity of tracts in the frontal lobe, parietal lobe, and corpus callosum (Nusbaum et al., 2001; O'Sullivan et al., 2001). Finally, aging demonstrates changes in mineral content, as indicated by microhemorrhages measured via MR-gradient echo imaging (Cordonnier et al., 2010), and changes in the axonal microstructure of brain tissue, as measured by magnetic resonance elastography (Arani et al., 2015). Structural neuroimaging techniques can provide a precise index of brain health by measuring the extent of changes in brain structure associated with healthy aging.

As a precise method of measuring age-related changes in the brain, structural neuroimaging is vital to the development of predictive dietary patterns of brain aging. Indeed, several structural aspects of brain health have been shown to be amenable to diet, indicating that neuroimaging can be a useful tool to characterize the relationship between nutrition and brain aging (Table 2). However, research in this area stands to benefit from the examination of nutrient biomarkers of dietary intake, along with the assessment of established dietary patterns, such as the MEDI diet (Willett et al., 1995), and the application of data-driven methods to further investigate the interactive nutrient combinations that are associated with healthy brain aging.

**Table 2:** **Summary of evidence examining the role of nutrition in structural changes associated with brain aging**.

**Structural component**	**Dietary component**
**WHOLE BRAIN MEASURES**	
Brain volume	Docosahexaenoic acid (Tan et al., 2012)
	Mediterranean diet (Gu et al., 2015)
	Vitamin E (Mangialasche et al., 2013)
	Vitamin C (Whalley et al., 2003)
Cortical thickness	Vitamin D (Walhovd et al., 2014)
	Mediterranean diet (Gu et al., 2015)
	Vitamin E (Mangialasche et al., 2013)
**REGIONAL MEASURES**	
Temporal cortex volume	Vitamin D (Hooshmand et al., 2014)
	Omega-3 polyunsaturated fats (Conklin et al., 2007)
	Eicosapentaenoic acid (Samieri et al., 2012)
Parietal cortex volume	Vitamin B6 (Erickson et al., 2008)
	Vitamin B12 (Erickson et al., 2008)
	Mediterranean diet (Gu et al., 2015)
Cingulate cortex volume	Vitamin B6 (Erickson et al., 2008)
	Omega-3 polyunsaturated fats (Conklin et al., 2007)
	Mediterranean diet (Gu et al., 2015)
Frontal cortex volume	Vitamin B6 (Erickson et al., 2008)
	Omega-3 polyunsaturated fats (Zamroziewicz et al., 2015)
	Mediterranean diet (Gu et al., 2015)
White matter lesions	Vitamin D (Annweiler et al., 2014)
	Vitamin B12 (de Lau et al., 2009)
	Docosahexaenoic acid (Tan et al., 2012)
	Choline (Poly et al., 2011)
	Mediterranean diet (Gardener et al., 2012)
	Marine omega-3 polyunsaturated fats (Bowman et al., 2012)
Intracerebral hemorrhage volume	Calcium (Inoue et al., 2013)

### Functional neuroimaging

**Figure 2:**
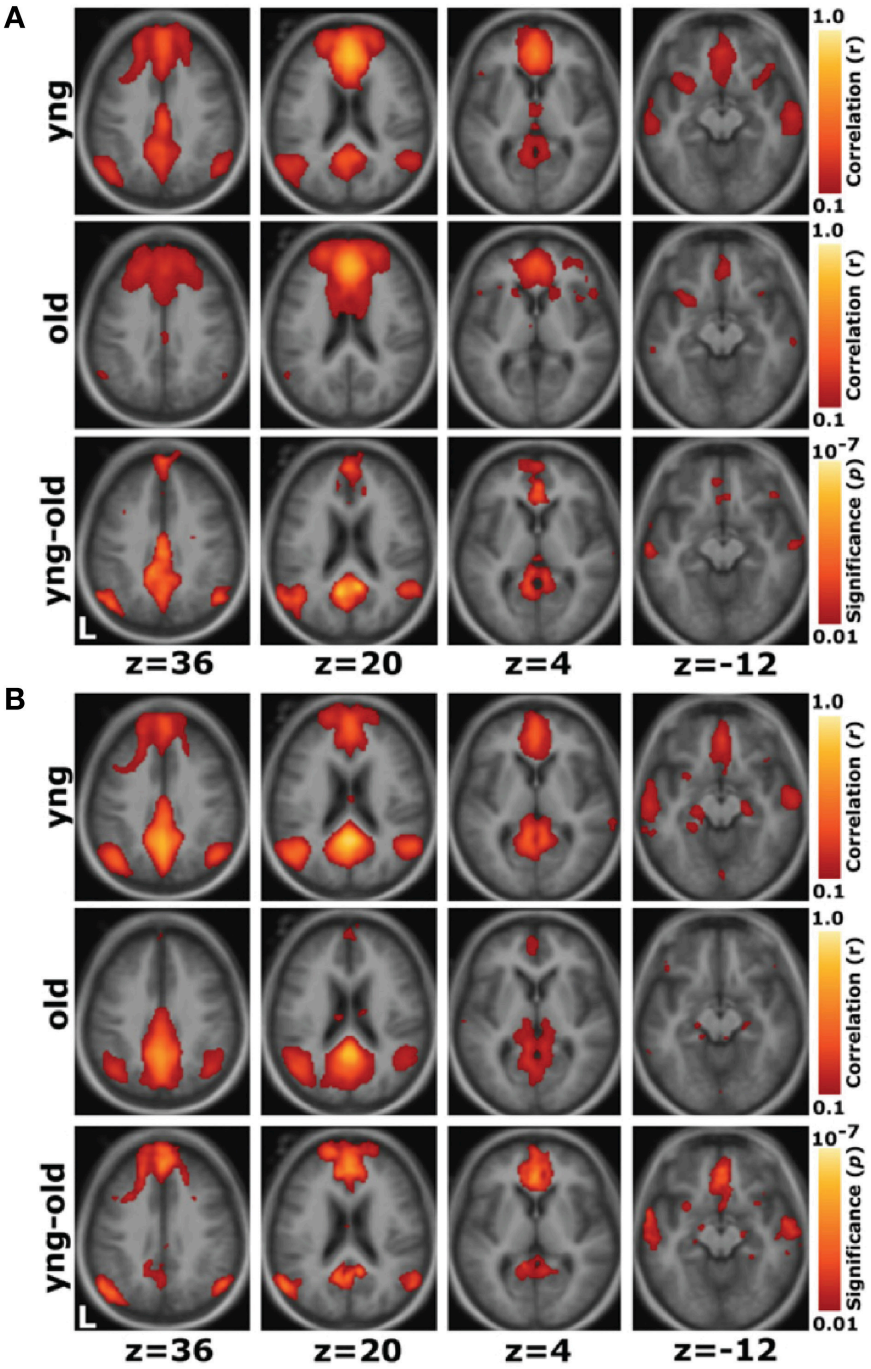
**Whole-brain exploratory analyses show reduced connectivity within the default network with aging. (A)** For a seed placed in the medial prefrontal cortex, positive correlations with the medial prefrontal cortex time course exceeding a threshold of *r* = 0.1 are colored in red to yellow and averaged for all young participants **(top)** and all old participants **(middle)**. A direct comparison of the two groups using the young-old contrast **(bottom)** highlights voxels at a significance level of *p* < 0.01. The young group shows higher correlations with many regions comprising the network. **(B)** The reverse scenario when a seed is placed in the posterior cingulate/retrosplenial cortex. Functional correlations between the posterior cingulate/retrosplenial cortex and both the medial prefrontal cortex and the bilateral lateral parietal cortex, as well as some hint of the hippocampal formation, decline in old age. Reprinted with permission from Andrews-Hanna et al. (2007).

Functional neuroimaging methods enable the investigation of functional brain changes that are associated with cognitive aging. Functional magnetic resonance imaging (fMRI) measures the ratio of oxygenated to deoxygenated hemoglobin in the blood as a marker of change in neural activity related to cognitively demanding tasks or rest (Lockhart and DeCarli, 2014). Functional neuroimaging has demonstrated that age-related decline in cognitive processes begins early—even when the prevalence of concomitant disease is low (Park and Reuter-Lorenz, 2009). These changes in brain activity are known to reflect alterations in underlying neurotransmission and brain structure that are concentrated in the prefrontal and temporal cortices (Tomasi and Volkow, 2012). Age-related changes in brain activity are characterized by greater activity in prefrontal cortical regions and weaker activity in posterior regions (see the posterior-anterior shift theory, Davis et al., 2008; Stuss and Knight, 2013), as well as reduced asymmetry in activity of the prefrontal cortex (see the HAROLD model, Cabeza, 2002). Furthermore, functional connectivity analyses indicate that rather than changing interactions across lobes of the brain in a homogenous way, aging has the strongest effects on interactions between regions that work together as networks (Lockhart and DeCarli, 2014). One network that demonstrates age-related changes is the default mode network, consisting primarily of regions within the medial prefrontal cortex, the posterior cingulate cortex, and the precuneus cortex. This network is active when an individual is awake and alert (i.e., during the “default mode”) but is not engaged during cognitively demanding, goal-directed tasks (Shulman et al., 1997; Raichle et al., 2001; Greicius et al., 2003) Funtional connections between regions within the default mode network are reduced with aging, suggesting that regions that work together become more weakly coupled in the aging brain (Figure 2; Andrews-Hanna et al., 2007). Thus, fMRI provides a powerful tool to investigate age-related changes in functional brain connectivity and may be applied to forecast the trajectory of cognitive decline in the aging brain (Park and Reuter-Lorenz, 2009; Salthouse, 2009).

The use of fMRI methods to characterize the relationship between diet and brain function, particularly in the context of brain aging, has been limited. The handful of studies published on this topic indicate that supplementation of omega-3 PUFAs modulates neural activity during tasks of working memory and calculation (Konagai et al., 2013; Boespflug et al., 2015). These findings motivate the investigation of how other nutrients and nutrient patterns may benefit age-related changes in brain function. Although emerging evidence suggests age-related changes in brain function are amenable to diet, the mechanisms through which nutrients influence brain function remain largely unexplored. Thus, integrating contemporary methods from nutritional epidemiology and neuroscience to examine the effects of nutrition on healthy brain aging remains a promising area for future investigation.

## An interdisciplinary approach to studying nutrition's impact on healthy brain aging

**Figure 3:**
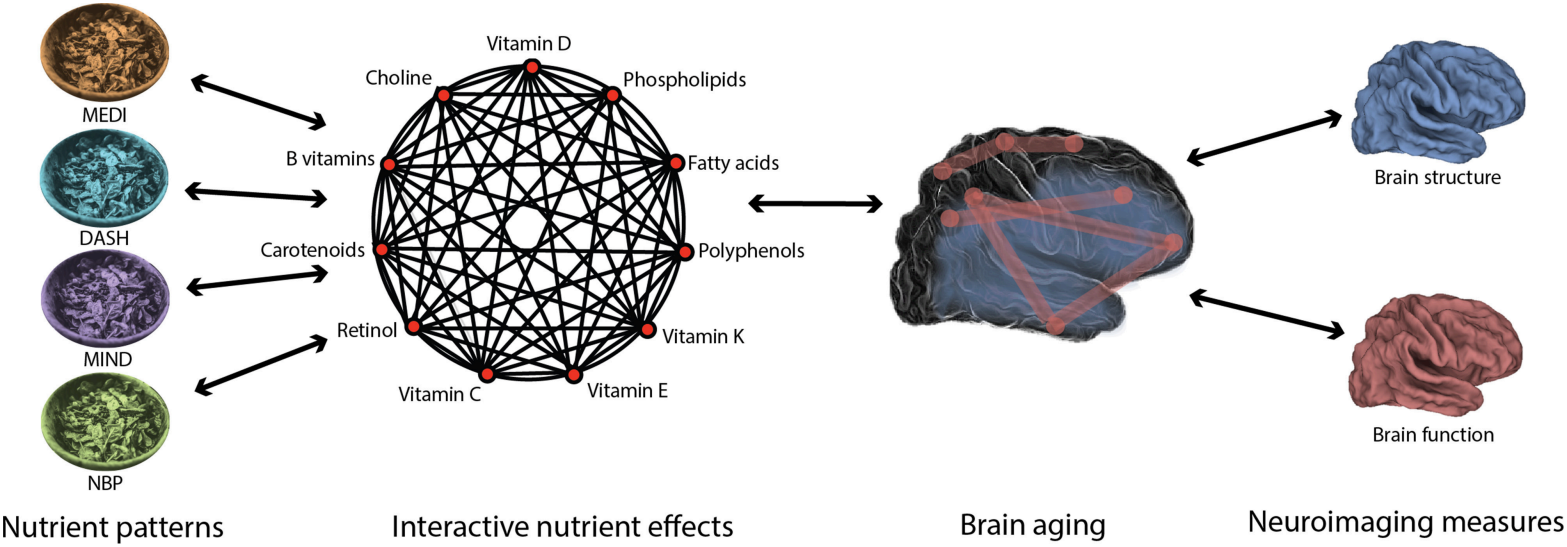
**Diet and brain aging are multifaceted in nature**. The interactive effects of nutrients in the diet may be captured using nutrient patterns, such as the Mediterranean diet (MEDI; Willett et al., 1995), the Dietary Approach to Stop Hypertension (DASH; Smith et al., 2010), the Mediterranean-Dietary Approach to Systolic Hypertension Diet (MIND; Morris et al., 2015), and Nutrient Biomarker Patterns (NBPs; Bowman et al., 2012). Likewise, the widespread changes in brain structure and function associated with age may be best measured using high-resolution neuroimaging methods. In order to understand the beneficial effects of nutrition on the aging brain, each of these complex entities must be characterized using precise methods.

Accumulating evidence indicates that the effects of nutrition on brain health are complex and multifactorial, reflecting the influence of particular nutrient combinations on specific aspects of brain aging. Indeed, nutritional epidemiology has shown that diets are composed of many nutrients that have interactive effects. This field has developed methods for deriving nutrient patterns (a priori hypothesized nutrient patterns such as MEDI and data-driven analyses such as NBPs) that capture the robust effects of nutrient interactions. Furthermore, cognitive neuroscience has shown that brain aging is a heterogeneous process characterized by widespread changes in structure and function. This field has developed neuroimaging methods to measure these changes with high-resolution. Predictive nutrient patterns of healthy brain aging will emerge from the integration of methods that sensitively capture variability in both diet and brain aging (Figure 3).

Recent findings from Zamroziewicz et al. (2015) indicate that gray matter volume of the anterior cingulate cortex mediates the relationship between omega-3 PUFAs and executive functions, demonstrating that the beneficial effects of nutrition on cognitive performance are mediated by cortical volume within specific brain regions (Zamroziewicz et al., 2015). Thus, this finding provides an example of how an interdisciplinary approach may be applied to study nutrition's impact on cognitive performance and brain health.

Research at the frontiers of nutritional cognitive neuroscience seeks to establish a personalized approach to **nutritional interventions** that takes into account individual variability in nutritional status and brain health. The goal of personalized nutrition is to enhance the precision of nutritional intervention and to enable novel applications to psychological health, aging, and disease. To achieve this goal, considerably more research is needed to elucidate the complex interactions within nutrient patterns, within processes of brain aging, and finally, between nutrient patterns and brain aging. Unanswered questions to guide future research are as follows:

1. How do the individual interactions between nutrients within dietary patterns benefit the aging brain?
2. How does age-related structural decline relate to changes in functional activity, and what mechanisms underlie associated declines in cognition?
3. How can variability in both diet and brain aging be captured and linked to build predictive patterns of healthy brain aging in precise and comprehensive ways?
4. How might known moderating variables—including age, genes, environment, and lifestyle—determine nutrition's impact on cognitive function and brain health?

KEY CONCEPT 4. Nutritional interventions By integrating methods from nutritional epidemiology and cognitive neuroscience, nutritional cognitive neuroscience seeks to elucidate nutrition's impact on the aging brain and to drive innovation in the design of nutritional interventions that promote healthy brain aging.

## Conclusion

Recent innovations in nutritional cognitive neuroscience hold promise for addressing the problems posed by a rapidly changing demographic landscape. This *Focused Review* highlights recent advances in nutritional cognitive neuroscience, with a focus on methods that investigate nutrient biomarkers that predict healthy aging. By applying cutting-edge techniques from nutritional epidemiology (nutrient biomarkers in *a priori* hypothesized dietary patterns and data-driven methods) and cognitive neuroscience (high resolution MRI measures of brain structure and function), the burgeoning field of nutritional cognitive neuroscience will continue to advance our understanding of the beneficial effects of nutrition on the aging brain. Ultimately, the development of predictive nutrient patterns for healthy brain aging will provide an empirically sound foundation for developing nutritional therapies that support the targeted treatment of cognitive and neurological impairments in the aging brain.

## Author contributions

MZ is the primary author of this review. AB is the primary investigator and contributed to drafting and editing of the manuscript.

### Conflict of interest statement

The authors declare that the research was conducted in the absence of any commercial or financial relationships that could be construed as a potential conflict of interest.
